# Current Concepts of Stem Cell Therapy for Chronic Spinal Cord Injury

**DOI:** 10.3390/ijms22147435

**Published:** 2021-07-11

**Authors:** Hidenori Suzuki, Takashi Sakai

**Affiliations:** Department of Orthopedics Surgery, Yamaguchi University Graduate School of Medicine, Yamaguchi 755-8505, Japan; cozy@yamaguchi-u.ac.jp

**Keywords:** chronic spinal cord injury, stem cells, glial scar, chondroitin sulfate proteoglycans, regenerative medicine, clinical trial

## Abstract

Chronic spinal cord injury (SCI) is a catastrophic condition associated with significant neurological deficit and social and financial burdens. It is currently being managed symptomatically with no real therapeutic strategies available. In recent years, a number of innovative regenerative strategies have emerged and have been continuously investigated in clinical trials. In addition, several more are coming down the translational pipeline. Among ongoing and completed trials are those reporting the use of mesenchymal stem cells, neural stem/progenitor cells, induced pluripotent stem cells, olfactory ensheathing cells, and Schwann cells. The advancements in stem cell technology, combined with the powerful neuroimaging modalities, can now accelerate the pathway of promising novel therapeutic strategies from bench to bedside. Various combinations of different molecular therapies have been combined with supportive scaffolds to facilitate favorable cell–material interactions. In this review, we summarized some of the most recent insights into the preclinical and clinical studies using stem cells and other supportive drugs to unlock the microenvironment in chronic SCI to treat patients with this condition. Successful future therapies will require these stem cells and other synergistic approaches to address the persistent barriers to regeneration, including glial scarring, loss of structural framework, and immunorejection.

## 1. Introduction

Spinal cord injuries (SCI) are a devastating event and can lead to physical, psychosocial, and vocational implications for patients and their family. In the United States, approximately 288,000 individuals are estimated to suffer from symptoms caused by SCI, and a recent survey showed the annual incidence of SCI to be approximately 54 cases per one million people [1,2]. Worldwide, the estimated incidence of SCI ranges from 250,000–500,000 individuals per year [3]. The majority of neuroregenerative therapy has focused on treating patients in the acute or subacute periods. In the acute to subacute phase, salvageable neuronal cells may still exist and the glial scar has not yet been established [4,5,6,7]. Unfortunately, 95% of patients with SCI are in the chronic phase of their injury [8].

Despite this pressing need, one of the greatest challenges in developing an effective therapy for chronic SCI has been the inhibitory microenvironment of the injured spinal cord. After SCI, astrocytes activate, proliferate, and migrate to the perilesional region where they form a dense interwoven network of processes and deposit chondroitin sulfate proteoglycans (CSPGs) into the extracellular matrix. Dystrophic axons surround the injury epicenter and become trapped in the dense meshwork of scar tissue [9].

Various cell populations can be used for the treatment of chronic SCI. Concurrently, several clinical trials using stem cells are underway around the world [ClinicalTrials.gov. Available online: https://www.clinicaltrials.gov/ (accessed on 1 June 2021)]. Among them, exogenous neural stem cell (NSC) therapies are particularly promising as these cells have the potential to differentiate into all three neuroglial lineages (i.e., neurons, astrocytes, and oligodendrocytes) to regenerate neural circuits, remyelinate denuded axons, and provide trophic support to endogenous cells [7,9,10,11,12].

This review summarizes the most recent insights into the preclinical and clinical studies using stem cell and other combinatory therapy for the treatment of chronic SCI. The authors provide an overview of chronic SCI and consider the current aspects of clinical stem cell therapy.

## 2. Barriers to Regeneration and Pathophysiology of Chronic SCI

**Figure 1:**
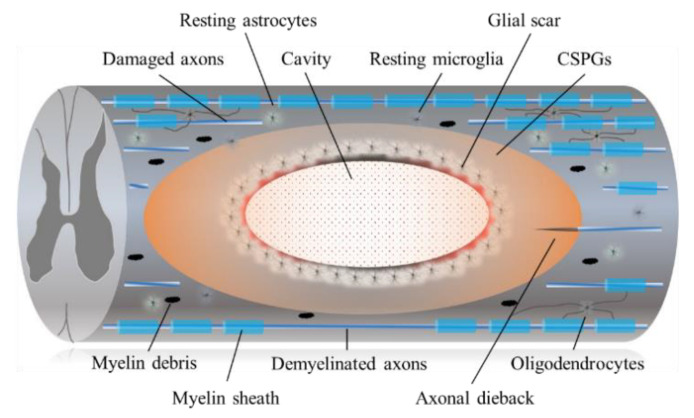
The diagram shows the pathophysiological events in the chronic SCI. Injury to the SCI results in death of neuronal as well as glial cells. Progressive demyelination results in degeneration of axonal fibers that leads to disruption of axo-glial signaling. A cavitation has occurred in the epicenter. Hypertrophic astrocytes with very long processes over the tips of non-regenerating fibers form a barrier known as a glial barrier or a glial wall around the cavitation. In response to damage/injury, microglial cells transform into active phagocytic microglia and exhibit chemotaxis (migrates and accumulates at the site of injury). The presence of CSPGs creates an inhibitory environment for axonal regeneration, which leads to failure of axonal growth cones at the injured site of CNS. In addition, CSPG also inhibits the migration and differentiation of oligodendrocyte progenitor cells.

It is widely recognized that regeneration of the adult mammalian central nervous system (CNS), including the spinal cord, is difficult due to its limited plasticity [13]. In the epicenter of a CNS lesion, a cavitation occurs that is surrounded by connective scar tissues containing cerebrospinal fluid. The phenotype of reactive astrocytes changes into scar-forming astrocytes that impede regenerating axons from crossing the lesion. Some inflammatory immune cells remain around the lesion even in the chronic phase of SCI (Figure 1). 

### 2.1. Astrocytic and Fibrotic Scar

Astrocytes proliferate and tightly interweave their extended processes around the perilesional region in an attempt to wall off the injury epicenter. Astrocytes, pericytes, and ependymal cells generate dense deposits of CSPGs as part of the fibrous scar, which bind leukocyte common antigen-related receptors such as protein tyrosine phosphatases. This activates GTPase RhoA and its downstream effector, rho-associated protein kinase (ROCK), leading to the collapse of the axonal growth cone and regenerative failure [14,15,16,17]. C3 transferase, an enzyme derived from Clostridium botulinum, locks RhoA in the inactive state and thereby inhibits Rho signaling. C3 transferase has been shown to promote axonal outgrowth on inhibitory substrates, both in vitro and in vivo [18,19]. The SPinal Cord Injury Rho INhibition InvestiGation (SPRING) clinical trial is now underway (NCT02669849) for acute SCI [2,20].

### 2.2. CSPGs and Chondroitinase ABC (ChABC)

CSPGs are a class of extracellular matrix molecule proteoglycans that are widely expressed within the CNS and that can be synthesized by all neural cell types [21]. CSPGs are highly upregulated in the glial scar after injury to the nervous system. In addition, they are mostly inhibitory and have been shown to hinder regeneration of axons across lesions in chronic SCI [22]. ChABC is a bacterial enzyme shown to effectively degrade CSPGs, including NG2, and to promote functional gains in mouse models after intrathecal administration [23,24]. Evidence also shows that co-administration of ChABC with neural precursor cells enhances transplant survival and remyelination of host axons [25,26]. More recently, large-scale CSPG digestion by direct lentiviral ChABC gene delivery into rat spinal cords resulted in a reduced cavitation volume and an enhanced preservation of axons. Treated rats also displayed an improved sensorimotor function on behavioral and electrophysiological assessments [27]. We also reported that ChABC administration reduced chronic injury scar and resulted in significantly improved NSCs derived from induced pluripotent stem cell (iPSC-NSC) survival with clear differentiation into all three neuroglial lineages. The chronically injured spinal cord can be ‘unlocked’ by ChABC pretreatment to produce a microenvironment conducive to regenerative iPSC therapy [9]. ChABC is an exciting therapy for which the optimal delivery modality remains to be elucidated. Future avenues of chronic SCI research may include exploration of human CNS-specific analogs of ChABC and its development.

## 3. Cell-Based Therapies and Characteristics of Stem Cells

At the lesion of the injured spinal cord, the death of the neuronal and neuroglial cells that make up the neural circuitry, along with the loss of cells tasked with its maintenance, is a main cause of functional impairment. The mechanism of cell death after SCI was characterized as an initial wave of necrosis at the lesion epicenter followed by a delayed phase of cell death in the neighboring tissue through necrotic and apoptotic mechanisms [28].

A stem cell is defined by its ability of self-renewal and its totipotency. The pluripotent stem cell differentiates into a multipotent cell of the three germ layers. In the field of regeneration therapy for SCI, adult stem cells, over embryonic stem cells, have an important advantage ethically because they present in adult, children, and umbilical cords. They can be harvested without destruction of an embryo [4,6].

Transplantation of NSCs, mesenchymal stem cells (MSCs) and Schwann cells harvested from various tissues, including iPSC, were reported as a cell therapy for chronic SCI [4,9,10]. We describe the characteristics of the stem cells for cell therapy on the details in Section 4 and Section 5.

**Figure 2:**
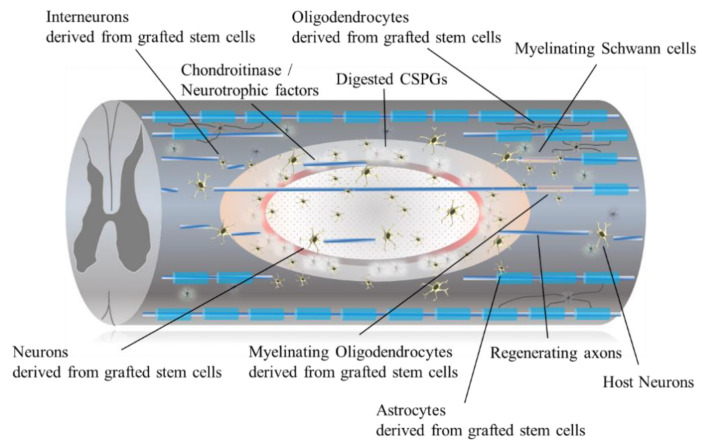
The diagram shows the pathophysiological change following stem cell transplantation. The transplanted stem cells differentiate into neural cells of the three lineages: neurons, astrocytes, and oligodendrocytes. Neurons and interneurons derived from grafted stem cells form new synaptic circuits and connectivity between host neurons and axons. Grafted stem cells secrete neurotrophic factors that improve morphological and behavioral outcomes after experimental SCI. Oligodendrocytes derived from grafted stem cells remyelinate damaged host axons. Regenerated and remyelinated axons pass throw the injured lesion and connect to other host neurons supported by interneurons and glial cells derived from grafted stem cells.

**Figure 3:**
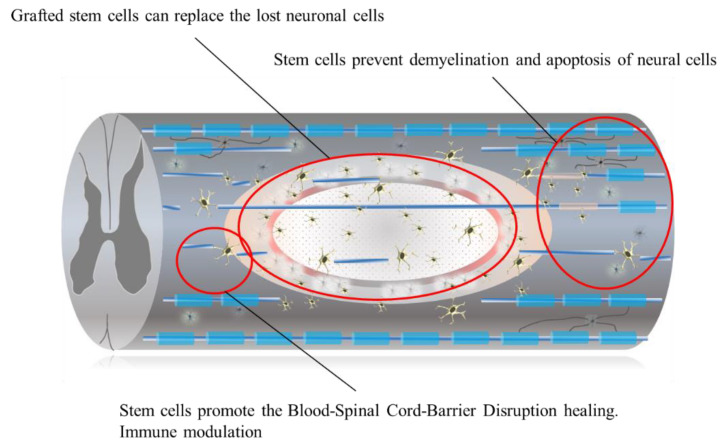
Grafted stem cells can replace the lost neurons, oligodendrocyte, and astrocytes. They prevent demyelination and apoptosis of neural cells they can modulate the immune response and also promote the blood–spinal cord barrier disruption healing.

Transplantation of the cells into the injured spinal cord is an obvious strategy to treat chronic SCI for repopulating cells that are not replenished by the endogenous regenerative process. Previous reports have revealed that engrafted cells work not only by repopulating cells but also by modulating the transplantation site into a more hospitable environment that prevents demyelination and apoptosis of neural cells [29] (Figure 2 and Figure 3).

We describe the mechanism of neurological recovery treated by each stem cell on the details in Section 4 and Section 5. 

Cell-based translational therapies have been attempted using several types of stem cells to induce nerve–axon sprouting or to neutralize the growth inhibitor factors. We show the ongoing clinical trials [ClinicalTrials.gov. Available online: https://www.clinicaltrials.gov/ (accessed on 1 June 2021)] currently targeting chronic SCI in Table 1 and excellent candidates for future clinical applications, given their promising preclinical results, in Table 2. 

## 4. Ongoing Clinical Trials in Chronic SCI

In this section, we focus on the stem cell therapies being investigated in the ongoing chronic SCI clinical trials. In addition, we show several important completed clinical trials. 

### 4.1. Umbilical Cord Mesenchymal Stem Cells

Mesenchymal stem cells (MSCs) are a promising tool for cell therapy. The umbilical cord (UC) is a good source of MSCs because their collection is noninvasive and the cells from this source are more capable and prolific than those obtained from other sites. It has been proven that differentiation, migration, and the protective properties of UC-MSCs are superior compared with those of other kinds of stem cells [62].

Recent studies suggested that the therapeutic effect of MSCs are mostly due to their paracrine activities. MSCs secrete GM-CSF, TGF-β, IGF-I, HGF, and stanniocalcin-1 which protect the death of survived neurons and oligodendrocytes [63,64,65,66], as well as IL-6, VEGF, PIGF, and MCP-1, which are relating angiogenesis [67]. In addition, MSCs secret the neurotrophic factors, glial-derived neurotrophic factors, brain-derived neurotrophic factors, and nerve growth factors [68]. These neurotrophic factors support the proliferation and regeneration of the remaining neurons [68]. MSCs exert their immunomodulatory effects via cell-to-cell contact and secretion of TGF-β, PGE-2, IL-10, galectin-1, indolamine 2, 3 dioxygenase, and HLA-G [65,69,70,71]. These data suggest that MSCs reduce the damages of the surrounding and remaining tissues by controlling the inflammation. MSCs also have antioxidant properties [72,73]. MSCs have therapeutic effects via direct cell fusion, mitochondrial transfer, and the production of microvesicles [73]. MSCs can inhibit gliosis, thus improving the ECM environment for better neurite growth [74].

Previous papers have reported the treatment of UC-MSCs in 34 chronic phase SCI patients (time from injury to participation 12–72 months) with AIS grade A. They revealed that 7/10 patients that received cell therapy demonstrated improvements in sensation, motion, muscle tension, and self-care ability, whereas only 5/14 patients in the rehabilitation group and 0/10 patients in the untreated control group showed improvement. Besides, the UC-MSC-treated patients also showed significant improvement in maximum bladder capacity and maximum detrusor pressure [87].

NCT03979742: This trial is evaluating the transplantation of UC blood mononuclear stem cells in combination with a 6-week course of oral lithium carbonate (Li_2_CO_3_) followed by intensive locomotor training for up to 6 h a day, 6 days a week, for 3–6 months to treat patients with chronic, stable, and complete SCI.

NCT03521323/NCT03505034: This trial aims to evaluate the safety and efficacy of intrathecal transplantation of allogeneic UC-derived MSCs for the treatment of different phrases of SCI. The history of SCI is divided into three periods, sub-acute SCI, early stage of chronic SCI, and late stage of chronic SCI, which, respectively, span the periods from 2 weeks–2 months, 2 months–12 months, and more than 12 months after injury.

### 4.2. Bone Marrow-Derived Mesenchymal Stem Cells

Bone marrow-derived MSCs/stromal cells are multipotent tissue stem cells that have shown promise in facilitating regeneration and/or recovery after neuronal injury [54,55].

Nogo-A, oligodendrocyte myelin glycoprotein (OMgp), and myelin-associated glycoprotein (MAG) are well known as myelin-associated proteins that inhibit axon growth and axonal regeneration in SCI. Bone marrow-derived MSCs stimulate neurite outgrowth over neural proteoglycans (CSPG), MAG and Nogo-A [56]. They described that MSC appeared to act as cellular bridges over the nerve-inhibitory molecules, perhaps directly masking them. The other papers revealed that spinal motor neurite outgrowth over glial scar inhibitors is enhanced by co-culture with bone marrow-derived MSCs that synthesize a number of cytokines and other neuroregulatory molecules, including brain-derived neurotrophic factor, nerve growth factor, stromal cell-derived factor 1, and vascular endothelial growth factor [57,58].

The Japanese Ministry of Health, Labor, and Welfare approved the sale of autologous MSC transplantation to patients with SCI in 2018 for the first time in the world. They reported the safety and feasibility following infusion of autologous expanded MSCs in subacute SCI patients. They also provided initial data that suggests rapid functional improvements following MSC infusion [59]. However, several issues still remain [60].

NCT0300336: This is a phase I/IIa, randomized, double-blind, two-arm, two-dose administration, placebo-controlled, two-way crossover clinical trial in which 10 patients ranging from 18 to 65 years of age affected with chronic traumatic spinal cord will enter the study with the objectives of assessing the safety of, and obtaining efficacy data on, intrathecal administration of expanded Wharton’s jelly MSCs [61].

NCT02574585: The purpose of this trial is to analyze the safety and efficacy of autologous bone marrow MSC transplantation in patients with thoracolumbar chronic and complete SCI.

NCT02574572: This trial will analyze the safety and efficacy of autologous bone marrow MSC transplantation in patients with cervical chronic and complete SCI.

NCT02570932 (completed): The purpose of this study was to analyze the potential clinical efficacy of intrathecal administration in the subarachnoid space of in vitro-expanded autologous adult bone marrow MSCs in the treatment of patients with established chronic SCI [88].

NCT01676441: This phase II/III clinical trial is designed to evaluate the safety and efficacy of autologous MSCs transplanted directly into the injured spinal cord.

NCT02688062/NCT02688049 (completed): The purpose of these trials is to investigate the efficacy and safety of the NeuroRegen scaffold with bone marrow mononuclear cells on neurological recovery following chronic and complete SCI, compared to treatment with surgical intradural decompression and adhesiolysis only. Partial sensory and autonomic nervous functional were improved in some patients; however, motor functional recovery was not observed. MRI suggested that NeuroRegen scaffold implantation supported injured spinal cord continuity after treatment. No adverse symptoms associated with stem cell or functional scaffold implantation were observed during the 3-year follow-up period [89].

### 4.3. Human Mesenchymal and Hemopoietic Stem Cells: Neuro-Cells

As both MSCs and hemopoietic stem cells (HSCs) have been shown to have the ability to differentiate into a spectrum of adult cell populations, many studies have sought to examine either the combined or individual contributions, to repair in vivo in a model of injury, and to potentially capitalize on the relationship between the two cell populations that are known to exist [90]. The engraftment of CD34+ human HSCs efficiently produces neurons in the regenerating chicken embryo spinal cord [91], and the use of MSCs to form guiding strands in the injured spinal cord promotes recovery [92].

NCT04205019: This single-center, open-label study is investigating the safety of the single intrathecal administration of Neuro-Cells in 10 end-stage (chronic) traumatic SCI patients. SCI is a rare disease without a potential cure, and Neuro-Cells are autologous fresh stem cells containing product that modulates secondary inflammation post SCI (one batch/one patient).

### 4.4. Neural Stem/Progenitor Cells

Pre-clinical data have suggested beneficial and functional effects of cell replacement-based therapy using multipotent neural precursors derived from animal or human fetal CNS embryonic stem cells, or iPSCs for treatment of a variety of spinal neurodegenerative disorders [9,11,30,31,32,33,34,35,36,37,38]. Exogenous neural stem/progenitor cell (NSPC) therapies are particularly promising as these cells have the potential to differentiate into all three neuroglial lineages (i.e., neurons, astrocytes, and oligodendrocytes) to regenerate neural circuits, remyelinate denuded axons, and provide trophic support to endogenous cells [9,10,11,39,40,41,42,93,94,95,96,97,98]. Grafted NPSC/NPCs bridges relay information during the repair process and lead to neuronal connectivity [9,99,100]. Some papers have reported that robust corticospinal axon regeneration, functional synapse formation, and improved skilled forelimb function after cells graft into sites of SCI; however, these studies were in the subacute phase of SCI [7,43]. Other papers have revealed that neurons derived from transplanted cells formed functional synapses with host circuits on patch clamp analysis in chronic SCI [9]. These reports also mean that grafted human NPSC/NPCs can support rodent corticospinal axon regeneration, indicating conservation of cell–cell interactions across species that enable growth of this system [9,43]. We show the ongoing clinical trials below based on the effectiveness of NSPC treatment in preclinical studies.

The “Pathway study” [101,102] used NSPCs derived from fetal tissue for transplantation into recruited patients with chronic cervical SCI lesions. Unfortunately, the study had to be terminated due to results being deemed too moderate for the manufacturer, StemCell Inc., and given that funding for the study was required for its completion. Functional improvement in hand was noted in some recruited patients after transplantation [103].

PALISADEBIO [5800 Armada Drive, Suite 210 Carlsbad, CA 92008, USA; PALISADEBIO. Available online: https://www.palisadebio.com/ (accessed on 1 June 2021)] began a phase I safety trial (NCT01772810) at the University of California San Diego of NSI-566 NSC transplantation for chronic thoracic SCI in 2014 [4,30]. In several patients, one to two levels of neurological improvement were detected using the ISNCSCI motor and sensory scores. It was reported that the safety of NSI-566 transplantation into the SCI site and early signs of potential efficacy in three of the subjects warrant further exploration of NSI-566 cells in dose-escalation studies [30]. Although this safety trial lacks the statistical power or a control group needed to evaluate functional changes resulting from cell grafting, these are important clinical proof-of-concept steps on the path to widespread translation of cell therapies.

### 4.5. Intravenous Transplantation of Multilineage-Differentiating Stress Enduring (Muse) Cells in Japan

In Japan, one of the recent topics in stem cell therapy for SCI is the clinical trial evaluating a Muse cell-based product (CL2020) [Life Science Institute, Inc. Available online: https://www.lsii.co.jp/en/ (accessed on 1 June 2021)]. Although the trial criteria only cover the subacute phase of cervical SCI, we purposely discuss the CL2020 trial because Muse cells are a very promising cell source for chronic SCI as well.

Muse cells are a novel type of non-tumorigenic pluripotent stem cells that can differentiate into various kinds of cells in the body [104]. Muse cells are endogenous reparative stem cells distributed in the bone marrow, blood, and connective tissue of organs. Their advantageous characteristics are represented by low safety concerns and the non-necessity of gene introduction or induction of differentiation prior to administration. Further, a surgical procedure to deliver cells is not necessary because of their specific ability to accumulate at the site of damage after intravenous administration, thus enabling treatment of patients only by intravenous drip of a Muse cell preparation, one of the simplest and most expedient approaches [104,105,106,107].

In this clinical trial, the target of treatment is subacute cervical SCI patients, and the aim is to study the efficacy and safety of intravenous administration of CL2020. Our institution is also participating in this trial.

## 5. Other Cells with Therapeutic Potential for Chronic SCI

### 5.1. Induced Pluripotent Stem Cells

Yamanaka and his colleagues developed iPSCs [44,45], which is the most famous recent development in stem cell research. iPSCs exhibit characteristics similar to those of embryonic stem cells; therefore, iPSCs can enrich ectodermal neural-lineage cells with appropriate culture induction. Previous studies have shown the efficacy of neural precursor cell transplantation in animal SCI models, and several mechanisms of functional recovery have been noted [6,33,34]. Recently, iPSCs have become the most promising cell source for chronic SCI treatment [31,32,46]. In the decade since iPS technology was first established, substantial progress has been made in developing safer and more efficient reprograming techniques, but a few key challenges, such as tumorigenicity and host immune rejection, continue to remain [34,47,48].

Several papers showed the effectiveness of iPSCs-NSCs transplant intervention in rodent models of chronic SCI. Grafted iPSC-based cells introduced at the site of the chronic SCI led to the integration of material into the injured spinal cord, reduced cavitation, and supported survival of the graft cells, but did not result in a statistically significant improvement of locomotor recovery [49]. Treatment with human-iPSC-NSC/precursor cells led to significantly greater axonal regrowth, remyelination by host-derived glial cells, and integration with the host neural circuitry through inhibitory synapse formation in chronic SCI [33]. We reported the combinatory treatment of ChABC and iPSC-NSC transplantation in chronic SCI. We presented important proof-of-concept data that the chronically injured spinal cord can be ‘unlocked’ by ChABC pretreatment to produce a microenvironment conducive to regenerative iPSC-NSC therapy [9]. These findings help to contribute to the recovery of motor function without deterioration, even after transplantation in the chronic phase of SCI.

In Japan, researchers are currently planning to launch a first in-human clinical study of an iPSC-based cell transplantation intervention for subacute SCI. In addition, as the next step, they plan to move to chronic SCI treatment using iPSCs-NSCs [32]. Several issues have been pointed out in previous papers that relate to this clinical trial. Some studies tried to exclude genetically unstable/undifferentiated iPSCs-NSCs before transplantation [33,50,51]. Another study tried to remove or ablate all of the abnormal cells after graft transplantation [52,53]. In terms of improving the safety and efficacy of iPSC transplantation intervention, we expect that the ongoing clinical trial will lead to the favorable result of neurological recovery in the study patients with chronic SCI.

### 5.2. Adipose Tissue-Derived Mesenchymal Stem Cells

One case report described experimental treatment with the use of autologous adipose tissue-derived MSCs (ADSCs) in chronic posttraumatic SCI in a domestic ferret with paresis of the back legs [75]. A pilot clinical study reported that percutaneous intraspinal injection of allogeneic canine ADSCs in dogs with chronic SCI led to an evaluation of improved locomotion [76]. ADSCs produce neurotrophic factors and may protect against hypoxia-ischemia and prevent glutamate neurotoxicity [76,77]. In rodent models of acute SCI, local transplantation of ADSCs promoted nervous tissue protection and functional recovery [78,79,80,81]. Despite promising results obtained in preclinical studies of acute SCI, there are no reports in the available literature describing successful application of ADSCs for SCI treatment in clinical trials in chronic SCI.

### 5.3. Olfactory Ensheathing Cells

Olfactory ensheathing cells (OECs) are a type of ensheathing cell that possess the characteristics of both astrocytes and Schwann cells (SC); they originate from the olfactory substrate located at the first and second layer of the olfactory epithelium in the olfactory nerve and bulb [108]. Several groups reported the safety and efficacy of OEC transplantation in chronic SCI patients [82,108,109,110,111,112,113]. Moreover, there were no severe complications with OEC transplantation [109]. Useful reticular formation functions were observed, but several papers lacked appropriate outcome measures [109,110]. In clinical trials using a similar cell source, researchers revealed that olfactory mucosal autografts are feasible, relatively safe, and possibly beneficial to people with chronic SCI when combined with postoperative rehabilitation [111,112].

### 5.4. Schwann Cells

As a candidate cell for SCI treatment, SCs possess many desirable properties that are known to promote regeneration and repair after CNS injury through a variety of possible mechanisms [83,84]. A preliminary report showed that autologous SC transplantation was generally safe for a selected number of SCI patients but that it did not provide beneficial effects [82]. In the Phase 1 clinical trial, the use of a combination of SCs and MSCs was safe for clinical application of spinal cord regeneration. Although no significant improvement was seen after transplantation, some degree of spasticity and neuropathic pain was reported [85,86].

### 5.5. Biomaterial Scaffolds and Stem Cell Combinatory Treatment

Recently, several combinatory treatments for chronic SCI with stem cells, biomaterial scaffolds, and others were reported in rodent models and clinically [9,74,114,115,116,117,118]. One of the biggest challenges in chronic SCI regeneration is to create an artificial scaffold that can mimic the extracellular matrix and support nervous system regeneration [119]. The prominent bio-scaffolds used for the SCI model include collagen, chitosan, fibrin, and PLGA [120]. Bio-scaffolds, such as PLLA and PLGA, combined with different kinds of cells and/or drugs, performed potential roles in recovering the lost hindlimb locomotor functions following SCI [120,121,122,123].

Poly(ε-caprolactone) electrospun nanofibers have good mechanical strength but also a prolonged biodegradation profile, making them unsuitable for neural implantation where remaining scaffolds may hamper tissue regeneration [81]. In contrast, injectable scaffolds made of self-assembling peptides belonging to the hydrogel family show remarkable regenerative potential because of their good biocompatibility, tailorability for slow drug release, and, most importantly, their easy functionalization with bioactive motifs [88].

Few bio-scaffold-based treatments showed robust therapeutic effects on promoting motor axons, especially CSTs regeneration across the injury epicenter [121,122]. Alternatively, some motoneurons including serotonergic (5HT), choline acetyltransferase (ChAT), and tyrosine hydroxylase (TH)-positive neurons were reported to have generated in the lesion center by functional bioscaffolds implantation [121].

Some researchers demonstrated that nanoscaffold technology platforms can be combined with other neurogenic drugs, as well as stem cell therapeutic efforts currently in development. [123]

Many researchers tend to agree that a multi-disciplinary approach is needed to solve chronic SCI repair. In this point of view, combinatory treatment of stem cells and biological scaffolds is quite an important approach to chronic SCI treatment in the future. We think that, in the future, stem cell therapy with the support of functionalized electrospun nerve scaffolds could be a promising therapy to cure nerve diseases in chronic SCI patients.

## 6. Conclusions

Currently, numerous clinical and experimental studies have shown positive results in terms of functional improvement with stem cell treatment in chronic SCI. Various designs of chronic SCI trials need to be performed. However, human chronic SCI trials are not easily enforced because of some inherent limitations. First, comparison between treatment and control groups is difficult because of ethical aspects, and, in terms of safety and efficacy, the results of animal experiments cannot be directly applied to humans. Nevertheless, a lot of basic research and clinical trials of stem cell therapy have already been performed, and promising results have also been reported. We are convinced that stem cell therapy will provide the drastic treatment needed for chronic SCI patients in the near future.

## Figures and Tables

**Table 1:** The ongoing clinical trials currently targeting chronic SCI and important completed clinical trials [ClinicalTrials.gov. Available online: https://www.clinicaltrials.gov/ (accessed on 1 June 2021)].

Identifier	Study Title	Phase	Subjects (Participants)	Cell Therapy	Route of Administration	Combination
NCT03979742	Umbilical Cord Blood Cell Transplant Into Injured Spinal Cord With Lithium Carbonate or Placebo Followed by Locomotor Training	Phase 2	27	UC Blood Mononuclear Stem Cells	Transplant into injured spinal cord	Oral lithium carbonate Locomotor Training
NCT03521323	Intrathecal Transplantation of UC-MSC in Patients With Early Stage of Chronic Spinal Cord Injury	Phase 2	66	Umbilical Cord Mesenchymal Stem Cells	Intrathecal	
NCT03505034	Intrathecal Transplantation of UC-MSC in Patients With Late Stage of Chronic Spinal Cord Injury	Phase 2	43	Umbilical Cord Mesenchymal Stem Cells	Intrathecal	
NCT04213131	Efficacy and Safety of hUC-MSCs and hUCB-MSCs in the Treatment of Chronic Spinal Cord Injury	Not Applicable	42	hUC-MSCs and hUCB-MSCs	Transplant into injured spinal cord	
NCT04205019	Safety Stem Cells in Spinal Cord Injury	Phase 1	10	Neuro-Cells (Autologous Fresh Stem Cells Containing Product)	Intrathecal	
NCT01676441	Safety and Efficacy of Autologous Mesenchymal Stem Cells in Chronic Spinal Cord Injury	Phase 2 Phase 3	20	Autologous Mesenchymal Stem Cells	Injection into the intramedullary and intrathecal space	
NCT01393977	Difference Between Rehabilitation Therapy and Stem Cells Transplantation in Patients With Spinal Cord Injury in China	Phase 2	60	Autologous BMSCs	Intrathecal	Rehabilitation
NCT02688062	NeuroRegen Scaffold™ With Bone Marrow Mononuclear Cells Transplantation vs. IntraduralDecompression and Adhesiolysis in SCI	Phase 1 Phase 2	22	BMMCs	Transplant into injured spinal cord *(v.s. Surgical intradural decompression and adhesiolysis)*	NeuroRegen Scaffold
NCT02688049	NeuroRegen Scaffold™ Combined With Stem Cells for Chronic Spinal Cord Injury Repair	Phase 1 Phase 2	30	Mesenchymal Stem Cells/NSCs	Transplant into injured spinal cord	NeuroRegen scaffold
NCT02352077	NeuroRegen Scaffold™ With Stem Cells for Chronic Spinal Cord Injury Repair	Phase 1	30	Bone Marrow Mononuclear Cells/Mesenchymal Stem Cells	Transplant into injured spinal cord	NeuroRegen Scaffold
NCT01772810	Safety Study of Human Spinal Cord-derived Neural Stem Cell Transplantation for the Treatment of Chronic SCI	Phase 1	8	Human Spinal Cord derived NSCs	Surgical implantation	
NCT02574585	Autologous Mesenchymal Stem Cells Transplantation in Thoracolumbar Chronic and Complete Spinal Cord Injury Spinal Cord Injury	Phase 2	40	Autologous Mesenchymal Cells	Percutaneous injections	
NCT02574572	Autologous Mesenchymal Stem Cells Transplantation in Cervical Chronic and Complete Spinal Cord Injury	Phase 1	10	Autologous Mesenchymal Cells	Transplant into injured spinal cord	
NCT01676441	Safety and Efficacy of Autologous Mesenchymal Stem Cells in Chronic Spinal Cord Injury	Phase 2 Phase 3	20	Autologous Mesenchymal Stem Cells	Injection into the intramedullary and intrathecal space	
NCT01354483 *(Comleted)*	Umbilical Cord Blood Mononuclear Cell Transplant to Treat Chronic Spinal Cord Injury	Phase 1 Phase 2	20	Umbilical Cord Blood Mononuclear Cell	Transplant into injured spinal cord	Methylprednisolone Lithium Carbonate Tablet Rehabilitation
NCT01186679 *(Comleted)*	Safety and Efficacy of Autologous Bone Marrow Stem Cells in Treating Spinal Cord Injury	Phase 1 Phase 2	12	Autologous BMSCs	Intrathecal	laminectomy
NCT02152657 *(Comleted)*	Evaluation of Autologous Mesenchymal Stem Cell Transplantation in Chronic Spinal Cord Injury: a PilotStudy	Not Applicable	5	Mesenchymal Stem Cells	Percutaneous injection	
NCT01909154 *(Comleted)*	Safety Study of Local Administration of Autologous Bone Marrow Stromal Cells in Chronic Paraplegia	Phase 1	12	Mesenchymal Stromal Cells	Intrathecal	
NCT01873547 *(Comleted)*	Different Efficacy Between Rehabilitation Therapy and Stem Cells Transplantation in Patients With SCI in China	Phase 3	300	Umbilical Cord Mesenchymal Stem Cells	Intrathecal	
NCT03003364 *(Completed)*	Intrathecal Administration of Expanded Wharton’s Jelly Mesenchymal Stem Cells in Chronic Traumatic Spinal Cord Injury	Phase 1 Phase 2	10	Mesenchymal Stem Cells	Intrathecal	
NCT02354625 *(Completed)*	The Safety of ahSC in Chronic SCI With Rehabilitation	Phase 1	8	Autologous human Schwann cells	Transplant into injured spinal cord	Rehabilitation
NCT02570932 *(Comleted)*	Administration of Expanded Autologous Adult Bone Marrow Mesenchymal Cells in Established Chronic Spinal Cord Injuries	Phase 2	10	Autologous BMSCs	Intrathecal	

**Table 2:** Excellent candidates for future clinical applications, given their promising preclinical results.

Cells	Source	Advantages	Disadvantages	Results	References
Neural Stem Cells/Neural Precursor Cells	Central Nervous System iPSCs	Neuronal differentiation Remyelination Secretion of trophic factors Host cells survival	Immune rejection Tumorgenesis	Functional recovery Graft cells survival and neuronal cell differentiation Secretion of trophic factors Protection of host neuronal cells Axonal outgrowth through injured lesion Remyelination of host axons	[7,9,10,11,12,30,31,32,33,34,35,36,37,38,39,40,41,42,43]
Induced Pluripotent Stem Cells(iPSCs)	Skin, Blood, Umbilical Cord, Adipose Tissue	Long-term self-renewing Pluripotency Functional recovery Neuronal differentiation Remyelination Secretion of trophic factors Host cells survival No ethical issues Low risk of immune rejection			[6,9,31,32,33,34,35,44,45,46,47,48,49,50,51,52,53]
Bone Marrow Mesenchymal Stem Cells	Bone Marrow	Secretion of trophic factors Functional recovery Host cells survival Low risk of immune rejection Autologous transplants No ethical issues	Difficulty of neuronal differentiation Low cell survival rate	Functional recovery Repair of spinal cord injury Secretion of trophic factors Protection of host neuronal cells Axonal outgrowth Remyelination of host axons	[54,55,56,57,58,59,60,61]
Umbilical Cord Mesenchymal Stem Cells	Umbilical Cord				[62,63,64,65,66,67,68,69,70,71,72,73,74]
Adipose Mesenchymal Stem Cells	Fat				[75,76,77,78,79,80,81]
Schwann Cells	Nervous System	Remyelination Functional recovery Secretion of trophic factors	No differentiation into neurons and astrocytes	Functional recovery Remyelination of host axons Axonal outgrowth Repair of spinal cord injury Secretion of trophic factors Protection of host neuronal cells	[82,83,84,85,86]

## Data Availability

Not applicable.
